# The Virtuousness of Ethical Networks: How to Foster Virtuous Practices in Nonprofit Organizations

**DOI:** 10.1007/s10551-023-05326-y

**Published:** 2023-01-10

**Authors:** Giorgio Mion, Vania Vigolo, Angelo Bonfanti, Riccardo Tessari

**Affiliations:** Department of Business Administration, University of Verona, Via Cantarane, 24, 37129 Verona, Italy

**Keywords:** Virtue ethics, Ethical networks, Organizational virtuousness, Social alliances, Networking, Nonprofit organizations

## Abstract

Ethical networks are an emerging form of social alliance based on collaboration between organizations that share a common ethical commitment. Grounded in a theoretical framework of virtue-based business ethics and focusing on nonprofit alliances, this study investigates the virtuousness of ethical networks; that is, how they trigger virtuous practices in their member nonprofit organizations. Adopting a qualitative grounded theory approach, the study focuses on one of the largest Italian ethical networks of nonprofit organizations operating in the social care sector. The findings show that shared ethical values and religious beliefs are positively associated with ethical network building. Based on these findings, a circular model of virtuousness is proposed in which ethical networks foster virtuous practices among their members at four levels: (1) the strategic orientation level, (2) the institutional level, (3) the organizational level, and (4) the relational level. At each of these levels, ethical networks foster a habituation to virtues and the propagation of virtuous behaviors among their members. Theoretical, practical, and social implications of the research findings are discussed.

## Introduction

The prevalence of unpredictable disruptive events such as the global financial crisis of 2008, the COVID-19 pandemic, and geopolitical instability in central Europe illustrates the importance of individuals and organizations practicing virtue ethics over utilitarian behaviors. Specifically, virtue ethics can help prevent and resolve crises by improving diagnostic instruments, prescribing more effective solutions (Moore, 2012a), reducing misbehaviors, and promoting good behaviors among individuals and organizations (Caza et al., 2004. The positive effects of virtue ethics occur when individuals cultivate their moral character and act ethically by practicing virtues in concrete life situations. Practical wisdom (*phronēsis* in Greek) as an intellectual virtue, along with character virtues such as courage and generosity, can enforce human strengths (Crossan et al., 2013) and correct weaknesses (Foot, 1979). By developing virtues, individuals become capable of making good decisions, even in complex situations. Practical wisdom, which implies the presence of all other character virtues, is the attribute of being a ‘good’ decision-maker, one who can make the right choices for themselves and the community.

Practical wisdom can be implemented in all human fields, including management (Intezari & Pauleen, 2014; Kinsella & Pitman, 2012; Puleen & Kupers, 2013; Schwartz, 2011). Therefore, organizations can pursue virtuousness. More precisely, virtuous organizations make it possible for individuals to practice virtues (Moore, 2015) by cultivating a collaborative and harmonic workplace (Morales-Sánchez & Cabello-Medina, 2015) and fostering cooperative relationships within and beyond organizational borders (Tsoukas, 2018). This last aspect highlights the fact that not only individual organizations (Bright, 2006; Caza et al., 2004) but also networks can be virtuous (Vriens et al., 2018). Ethical networks involve various business or cross-sector alliances that cooperate with each other (e.g., Arya & Salk, 2012; Liu et al., 2018; Selsky & Parker, 2005; Silvestri & Veltri, 2017) in emphasizing the advancement and enactment of ethical standards (Burchielli et al., 2009).

The extant research on virtue ethics in ethical networks is limited; thus, this topic requires further study. Previous scholars have aimed to understand these organizational forms in general terms; for example, the types (Melé, 2009b) and advantages (Silvestri & Veltri, 2017; Vaccaro, 2012) of networking or the conditions needed to be virtuous (Vriens et al., 2018), such as the dynamics needed to create sustainability innovations (Dossa & Kaeufer, 2014) or the impact of blockchain technology on ethical work environments (Sharif & Ghodoosi, 2022). Some authors have only theoretically highlighted the importance of fostering virtues within networks such as by promoting a common purpose and shared ethical vision and values, fostering cooperation between individuals, and overcoming narrow, self-interested human behaviors (e.g., Vriens et al., 2018). Therefore, to the best of our knowledge, little research on virtuous ethical networks has been conducted to understand the development of virtuous practices within individual organizations belonging to the same ethical network. Past studies have examined this topic only with reference to organizations not belonging to a network (e.g., Cameron et al., 2004; Meyer, 2018; Sison et al., 2018). Given that the virtuousness of an ethical network can facilitate the flourishing of virtues in organizations belonging to that network, it is pertinent to understand from an ethical network perspective how individual behaviors develop in organizations. Therefore, the following research question emerges:

RQ: *How do ethical networks foster virtuous practices among their members?*

Adopting a virtue-based business ethics framework and focusing on nonprofit alliances, this study aims to investigate the virtuousness of ethical networks; that is, to explore how they promote virtuous practices within their member nonprofit organizations. This question is important in management research because virtues operate not on a theoretical level but in practical situations in which goals, context, and personal characteristics are essential (Sison et al., 2018). In addition, this research considers the perspectives of the network’s members, understood as organizational nodes of the network, and examines the virtuousness of the network from the eyes of those who experience it daily, thus providing a vision of the network from within the network itself. Further, members’ perspectives can help in understanding the actual impact of membership to an ethical network, thus contributing to the debate on the virtuousness of organizations from a descriptive/empirical point of view.

In methodological terms, this study adopts a qualitative method by focusing on a single case study, an Italian ethical network of nonprofit organizations operating in the social care sector. The members of this network share ethical values inspired by a common religious belief. Based on Gioia’s (2013) methodology and grounded theory (Glaser & Strauss, 1967; Strauss & Corbin, 1998), this study proposes a model for the development of an ethical network that can foster virtuous behaviors both within the network and among its members. Despite its local setting, the case possesses unique characteristics relevant to the research question (Siggelkow, 2007; Stake, 1995) and may be valuable to future researchers investigating ethical networks in the social care sector in other parts of the world. Overall, the findings contribute to the broader academic and managerial debate on the role of ethical networks in supporting nonprofit organizations to virtuously pursue their ethical missions.

The remainder of the paper is organized as follows. The following section provides an overview of the theoretical background on virtue ethics theory, virtuous organizations, and ethical networks. The research setting and method are then described, followed by a presentation and discussion of the findings. The final section concludes the paper and discusses study limitations and future research directions.

## Theoretical Background

In the literature, virtue ethics is primarily a theoretical approach that is crucial for understanding individual behaviors in organizational practices (e.g., Sison et al., 2018). Previous studies also show that not only individual organizations but also networks can become virtuous to better serve individuals and communities (Bright, 2006; Caza et al., 2004; Vriens et al., 2018). However, research on the virtuousness of ethical networks is lacking. The challenge for researchers is to discover how an ethical network can foster virtuous practices across the individual organizations belonging to that network. This section begins by discussing virtue ethics theory, shedding light on moral virtues and practical wisdom as constituting a person’s moral character. This is followed by a discussion on virtuous organizations and organizational purpose as a crucial characteristic. Finally, we examine ethical networks and their virtuousness.

### Virtue Ethics Theory

Inspired by Aristotle’s *Nicomachean Ethics*, virtue ethics theory puts individuals and their ability to act well at the center of the discourse. This contrasts with utilitarianism and deontologism, which focus on utility and moral rules, respectively. Virtue ethics is considered a robust and workable approach to management that shifts attention from agents’ (managers’) economic utility (or external duties) to their moral character and ability to act for the good of both individuals and the community of which they are a part.

Following Sherman (1989), one’s moral character comprises virtues—both character virtues and the intellectual virtue of practical wisdom—that together enable one to make choices guided by the desire to do the right thing and the capacity to act in accordance with this desire. More precisely, moral virtue refers not to individual character traits that express the concept of virtue but rather to “a disposition to act, desire, and feel that involves the exercise of judgment and leads to a recognizable human excellence or instance of human flourishing” (Yearly, 1990, p. 13). Further, moral virtue is “an acquired disposition that is valued as part of the character of a morally good human being, and that is exhibited in the person’s habitual behavior” (Velasquez, 2002, p. 135) by including “permanent dispositions that favor ethical behaviors” (Melé, 2005, p. 101). These definitions emphasize that one’s moral character overcomes one’s individuality and that people have intellectual and spiritual lives and are naturally oriented toward non-self-interested relations (Acevedo, 2012; Alford, 2010). Thus, virtue-based ethics focuses on the ethical/moral agent (Melé, 2009a) by considering ethical behavior as the result of an individual’s moral character rather than arising from mere compliance with the rules defined by society or the institutions to which the individual belongs (Pincoffs, 1986).

In recent decades, scholars have focused on the possibility of applying a virtue ethics framework to management (e.g., Sison et al., 2018), reinvigorating the business ethics debate. From this perspective, virtue ethics refers to managers’ capacity to make wise decisions, both ethically and in terms of business success (Audi, 2012), given that virtuousness can stimulate performance (Cameron et al., 2004) and buffer against organizational dysfunction (Caza et al., 2004). For example, during the COVID-19 pandemic, both health personnel and volunteers in healthcare organizations were available to offer mutual assistance, even when the economic incentives did not align with the risks or when there was no economic compensation at all (e.g., Mion et al., 2021). Hence, virtuous behavior does not mean making the same decision in response to every circumstance but rather requires one to consider the specific context in which the decision is being made. While all agents make decisions, they become moral agents when exhibiting a value structure and pursuing moral commitments in their practices (Sayer, 2011; Selznick, 2016). Previous authors have defined these practices as a form of cooperative activity in which a continuous striving toward moral excellence enhances the human capacity for excellence and raises awareness of the good that can result (MacIntyre, 1985). Beabout (2012) argues that to achieve excellence, managers should develop the intellectual and moral virtue of practical wisdom. Practical wisdom is at the center of virtue ethics because it facilitates the relationship between individual practices/decisions and organizational purpose. Practical wisdom is essential for balancing that which is ‘right’ or ‘good’ for the decision-maker and that which is ‘right’ or ‘good’ for the community or organization. Therefore, a wise decision enhances the common good for both individuals and the community. Practical wisdom cannot be learned by studying; rather, it results from life experience and is fostered by cultivating social (Coleman, 1990; Melé, 2003) and spiritual (Lenssen et al., 2012) capital. People can cultivate social and spiritual capital throughout their social, affective, and intimate lives and in each living environment that enables them to build human relations, thus overcoming the mere exchange of interests. Individuals who experience a collaborative work environment can become more collaborative themselves, for instance, by improving their ability to understand other people’s needs if they are immersed in a community where solidarity and trust form the bases of internal and external relationships. Thus, practical wisdom is considered an enabler of virtuous organizations (Moore, 2015).

### Virtuous Organizations

An organization may be considered virtuous in proportion to its attitude to supporting the enhancement of its members’ moral character through virtuous practices (Cameron et al., 2004). Although previous authors have identified various factors characterizing the virtuousness of an organization (Bright et al., 2014), organizational virtuousness cannot be reduced to the sum of individual virtues; rather, it refers to the organizational characteristics that play a positive role in developing individual virtues and effective organizational relationships (Cameron et al., 2011). In addition, an organization can be considered virtuous if its purpose is to create “goods of first intent” (Bright, 2006, p. 753) and its orientation is toward excellence rather than success (Fernando & Moore, 2015; Moore, 2012b). According to Moore (2015), a virtuous organization is characterized by a “good purpose and appropriate ordering of success and excellence, both enabled by the intellectual virtue of practical wisdom.” (p. S108).

From these definitions, organizational purpose emerges as a crucial characteristic of any virtuous organization. After all, virtues are associated with the goals of action. However, it is not correct to state that “virtue makes the goal right” (Moss, 2011, p. 205); indeed, wise decision-makers are not only competent in technical and professional terms but also conscious of the purpose of the action and capable of making choices aimed at the good of both themselves and the community, overcoming a narrow individual vision of management. As affirmed by Melé (2009a), in contrast to individualism, “which understands society as being made up exclusively of mutual interests and social contracts, the Aristotelian–Personalist view of society considers interdependences and the existence of a ‘civic friendship’ as a base for justice” (p. 234). Therefore, there is a logical connection between individual practical wisdom, the organization, and its purpose. Even if the virtuous moral agent is an individual, virtues overcome the narrow boundaries of individual behaviors and concern the organizations in which individuals act for two reasons: virtue feeds on social capital, and the nature and mission of the organization can profoundly influence individual behavior.

The literature highlights the characteristics that make an organization virtuous. Specifically, a virtuous organization provides their members with three contexts (Vriens et al., 2018): (1) a teleological context regarding the purpose of the organization, its values, and the managerial and organizational tasks related to the (ethical) purpose; (2) a deliberative context, which is associated with the understanding of individual and organizational consequences of actions; and (3) a social context, which concerns an understanding of the social effects of organizational activities.

Virtuous organizations foster the flourishing of virtues by enhancing cooperation between individuals and promoting a common purpose and shared values. For example, training programs should focus not only on professional skills but also on relational skills, ethics, and motivational instruments (Melé, 2005).

### Ethical Networks

It is not only individual organizations that can be considered virtuous; a network can also be virtuous in the presence of specific conditions (Vriens et al., 2018) such as a clear ethical commitment and shared values. An ethical network may be considered analogous to the concept of a social alliance (Silvestri & Veltri, 2017); that is, as a form of collaboration between different types of businesses (e.g., alliances between for-profit and nonprofit organizations) (Liu et al., 2018) or cross-sector alliances (Arya & Salk, 2012; Selsky & Parker, 2005). The organizations belonging to an ethical network share a clear common ethical commitment—for example, supporting disadvantaged people or developing an underdeveloped area (Silvestri & Veltri, 2017; Vaccaro, 2012). As a result, starting from a common ethical framework (e.g., a religious belief), members of ethical networks share common objectives and pursue similar activities aimed at ethical changes and outcomes in society (Burchielli et al., 2009). The juridical or other institutional characteristics of the members of an ethical network are not crucial—for example, they may belong to different industries or be profit or nonprofit oriented—but they must share common values and beliefs.

The concept of the ethical network derives from the notion of social capital and refers to the social relationships—whether between individual people or individual organizations—and to the “norms of reciprocity and trustworthiness” (Putnam, 2000, p. 19) that arise from these relationships. The goodwill that binds network members makes it possible to share information, influence, and solidarity (Sandefur & Laumann, 1998). In a network derived from social relationships rather than from market or hierarchical relationships, objects of exchange are gifts or favors, and the terms of exchange are diffuse and less specific than those in commercial contracts (Adler & Kwon, 2002, p. 19). In an ethical network, the common ethical framework is at the center of the social structure from which the network itself derives. The nature of the social capital accumulated by network members assumes specific characteristics related to ethics. Adler and Kwon (2002) highlight that social capital could substitute for, or even complement, other forms of capital. That is, the building of trust between an ethical network’s members makes possible the development of common objectives and actions by enforcing relationships and community building (Edelman et al., 2004; Halpern, 2005).

Ethical risks may arise from the potential economic advantages of belonging to a network and sharing social capital (Ayios et al., 2014). However, ethical networks differ from other networks precisely because ethics are placed at the center of relationships, and the ontology of the network affects the ethical issues that may arise during its operation (Halinen & Jokela, 2016). Therefore, being grounded in ethics can limit the risks mentioned above.

Floridi (2009) asserts that by applying a network approach to business ethics, profits, rather than being the main goal, are simply a positive consequence of actions with a broader purpose, such as enhancing the common good. The network perspective encourages ethical behavior and enforces a commitment to common objectives, as affirmed by Haney (2017): “When managers view their firm as an integrated member of various networks and sub-networks, management processes tend to be collaborative and tend to focus on the sustainability of the system” (p. 270). Similarly, Silvestri and Veltri (2017) demonstrate how a network based on shared ethical values can achieve positive results for the benefit of the network itself as well as for its members, the local community, and other stakeholders. Nonetheless, previous studies have mainly focused on the impact of ethical networks on local communities and society in general, while empirical evidence on the effects of ethical networks on individual members of the alliance is lacking. Consequently, this study addresses this gap by investigating how ethical networks foster virtuous practices among their members.

## Research Methodology

### Research Design

For this study, under the ontological perspective, the researchers adopt a nominalist approach, according to which the social world is created through the interaction of actors. Epistemologically, they assume a strong constructionist perspective, which aims to providing “a rich picture of life and behavior in organizations or groups” (Easterby-Smith et al., 2015, p. 263). Based on grounded theory (Glaser & Strauss, 1967; Strauss & Corbin, 1998), a widely used method in business ethics research (e.g., Giuliani et al., 2021; Liedong, 2021; Liu et al., 2015) to understand the social constructions of research respondents (Charmaz, 2008), this study provides an in-depth analysis of a single case study (Beverland and Lindgreen, 2010; Eisenhardt, 1989). Specifically, it adopts an interpretive approach (Gioia et al., 2013; Nag and Gioia, 2012) in which concepts and themes that emerge from the data are developed to derive theoretical, practical, and social implications. This method balances the need for inductive concept development against that for systematic and rigorous analysis in qualitative research (Gioia et al., 2013). The choice to use a qualitative approach emerges from the desire to conduct an in-depth investigation of a phenomenon in its real-life context (Yin, 2015) and gather the perceptions of real people involved in such a reality (Lamnek, 2010). The case study method is well suited to the exploratory nature of the research question. Specifically, the single case study is considered the most appropriate methodology for exploratory research (Gibbert et al., 2008) to examine the ‘how’ of a little-known phenomenon (Yin, 2015) or for theory-building purposes (Eisenhardt, 1989).

### The Research Context

The selected case study is a network of 43 nonprofit organizations (foundations, associations, social cooperatives, ecclesiastical institutions, and other legal entities) operating in Verona, Italy, known as the Associazione Diocesana Opere Assistenziali (ADOA) (Diocesan Association of Helpful Organizations). ADOA unites value-driven organizations based on a joint ethical commitment (Silvestri & Veltri, 2017; Vaccaro, 2012). Thus, it responds to the characteristics of an ethical network as defined by Burchielli et al. (2009) because it shares with its members a common ethical framework while giving each member the freedom to operate and preserve its ethical mission. A preliminary analysis of organizational documents, including the ADOA Statute, strategic plans, and social reports, served to ensure that the case study selected would be suitable for answering the research question.

ADOA serves more than 20,000 people annually. The network has no direct employees, but its members employ 4082 staff and almost 500 volunteers. ADOA was founded in 2000 on the initiative of the Bishop of Verona and initially involved the four prominent nonprofit long-term care organizations of the diocese. The stated objective of the association is to preserve the charitable missions of nonprofit organizations through the flexible coordination of the activities of the associated entities (ADOA Statute, Article 2) and the maintenance of their statutory and patrimonial autonomy.

ADOA is unique in Italy in terms of its network characteristics, including its size (as one of the biggest), the ethical homogeneity of members (all share a Christian belief), the plurality of services offered (social care services to older people, people with physical and mental disabilities, and people with other needs), and longevity (22 years of age at the time of writing).

ADOA was developed around a common ethical framework based on Christian social thought. Article 5 of the ADOA Statute requires that members “operate in accordance to Christian principles... and the Social Doctrine of the Church in the fields of social care and healthcare sector.” According to the ADOA Statute, all members declare an ethical commitment and adherence to Christian values in their institutional documents (e.g., statutes and codes of ethics).

### Sampling and Data Collection

Purposeful sampling was used. More precisely, the ethical network board was contacted, informed about the research project, and asked to support the researchers in the data collection process, which included three direct observations, a focus group, and 22 in-depth interviews. These methods are all suitable for this type of research because direct observations are a nonintrusive qualitative method to understand a new phenomenon (e.g., Grove & Fisk, 1992), while focus groups and interviews enable researchers to obtain a wide range of ideas and perceptions about a specific phenomenon (e.g., Krueger & Casey, 2000). To demonstrate the trustworthiness of the research process (Cloutier & Ravasi, 2021), the phases of the research process are reported in Table 1, while details about data collection are described in Table 2. Specifically, to comply with the wishes of the ADOA board, the researcher did not record the three direct observations of the board meetings but took notes that helped to provide a deep understanding of the research context and its peculiarities. In addition, the direct observations enabled the identification of members who were particularly engaged in the ethical network and who would be suitable participants in the subsequent focus group.

**Table 1:** The phases of the research process

Research phases	Phase 1	Phase 2	Phase 3	Phase 4	Phase 5
Aim	Identification of a relevant case study to respond to the research question	Refinement of the research question and identification of relevant informants	Collection of informants’ voices	Protocol refinement and collection of informants’ voices	Abductive analysis (constant comparison between data and literature)
Activities	Preliminary analysis of online documents and literature	Observation Focus group Protocol development	Data collection (in-depth interviews) and preliminary analysis	Protocol refinement and further data collection (in-depth interviews) and analysis	Grounded theory articulation

**Table 2:** Data collection

Data collection method	Number	Participants	Duration	Data transcription
Direct observations	3	The Board of ADOA	1 Observation: overall 60 min 2 Observation: overall 40 min 3 Observation: overall 90 min	2 Pages 2 Pages 4 Pages
Focus group	1	5 Members of the Board of ADOA	Overall 120 min	5 Pages
Interviews	22	Managers and members of the Boards of the nonprofit organizations (see Table 3)	Minimum 40 and maximum 70 min (average 53 min)	Minimum 3 and maximum 7 pages (average 4 pages)

The focus group, which was conducted with five members of the ADOA board, was crucial to refine the research question and define the preliminary protocol for the in-depth interviews. The focus group lasted for 2 h and was moderated by one of the researchers. A second researcher was present to take notes.

The observations and focus groups enabled the researchers to identify the most relevant informants for the study. This study followed a grounded theory methodology in which the theory is grounded in the data and, most importantly, “in the informants’ experience and their understanding of that experience” (Gioia, 2021, p. 22). Therefore, while multiple data sources were used (see Table 2), the heart of this research lies in the semi-structured interviews conducted with “knowledgeable agents” (Gioia et al., 2013, p. 17) of the network.

To increase the richness of the data, interviewees included both managers and board members of the nonprofit organizations. All functional areas of ADOA (services for older people, services for people with disabilities, and charity services) were represented. Initially, 15 prospective participants were contacted via email and invited to participate in the interviews; all agreed to participate. Given that sampling in grounded theory is sequential, the researchers began with selective sampling before undertaking theoretical sampling once concepts began to emerge (Draucker et al., 2007; Gioia et al., 2013; Glaser & Strauss, 1967). Therefore, additional interviews were conducted until theoretical saturation (Glaser & Strauss, 1967) was reached (after seven interviews). In this process, prior informants were ‘backtracked’ to ask them questions that emerged in subsequent interviews (Gioia et al., 2013). Interviewees were not compensated for their participation. The interviewees’ profiles are reported in Table 3.

**Table 3:** Interviewees’ profile

Interviewees’ code	Position in the organization	Age	Gender	Area	Institutional form	Dimension	
						Employees	Volunteers
I1	Manager	36–45	Male	Elderly	Foundation	50–99	< 50
I2	Manager	56–65	Female	Elderly	Foundation	> 150	100–149
I3	Member of the board (President)	56–65	Female	Disabled	Cooperative	100–149	< 50
I4	Manager	36–45	Male	Disabled	Cooperative	100–149	< 50
I5	Member of the board	46–55	Male	Disabled	Foundation	< 50	< 50
I6	Manager	46–55	Male	Charity	Cooperative	< 50	100–149
I7	Member of the board	56–65	Female	Charity	Religious institution	> 150	< 50
I8	Manager	36–45	Male	Disabled	Association	< 50	50–99
I9	Manager	36–45	Male	Elderly	Foundation	50–99	< 50
I10	Member of the board	56–65	Female	Disabled	Association	< 50	50–99
I11	Manager	46–55	Male	Charity	Religious institution	50–99	> 150
I12	Member of the board	46–55	Male	Disabled	Association	< 50	> 150
I13	Manager	46–55	Female	Charity	Association	< 50	> 150
I14	Member of the board (President)	46–55	Male	Charity	Religious institution	50–99	> 150
I15	Manager	46–55	Male	Disabled	Religious institution	100–149	< 50
I16	Manager	36–45	Male	Education	Foundation	< 50	< 50
I17	Middle-manager	< 36	Female	Elderly	Foundation	50–99	< 50
I18	Middle-manager	36–45	Female	Disable	Religious institution	50–99	< 50
I19	Member of the board	36–45	Male	Disable	Foundation	< 50	< 50
I20	Member of the board	36–45	Male	Elderly	Association	< 50	< 50
I21	Middle-manager	46–55	Female	Elderly	Foundation	50–99	< 50
I22	Member of the board (President)	56–65	Male	Elderly	Foundation	50–99	< 50

The interviews were aimed at giving voice to the informants; hence, to represent their voices, direct quotations are reported in the results, as recommended by the Gioia methodology (Gioia, 2021; Gioia et al., 2013). Before conducting the interviews, careful attention was given to the initial interview protocol to ensure that it focused on the research question, was thorough, and did not contain “leading-the-witness questions” (Gioia et al., 2013, p. 19). As the research progressed, attention was also given to the revision of the protocol.

### Data Analysis

Data analysis and interpretation followed the approach recommended by Gioia et al. (2013); this helped prevent the loss of information by coding the informants’ voices (the data corpus) as first-order terms before aggregating them to second-order themes and, finally, identifying the aggregate dimensions. At the initial stage of data analysis, numerous informant terms, codes, and categories emerged. Gioia et al. (2013) describe this stage as a process similar to Strauss and Corbin’s (1998) notion of open coding. This first-order analysis attempts to faithfully adhere to informant terms to avoid “the theoretical arrogance that leads scholars to go overboard in imposing their ways of understanding on the informants” (Gioia et al., 2021, p. 24). Next, the researchers searched for similarities to reduce the number of key categories. In the second-order analysis, the researchers focused on emerging concepts that had not been adequately addressed in the literature (Gioia, 2013). This analysis implied continuous cycling between the data and the literature to assess whether the findings already had a theoretical basis or new concepts had been discovered. When no significant new concepts and themes emerged, the researchers concluded that theoretical saturation had been reached (Glaser & Strauss, 1967), and data collection was discontinued. The second-order themes were subsequently distilled into four aggregate dimensions (Gioia et al., 2013).

**Fig. 1:**
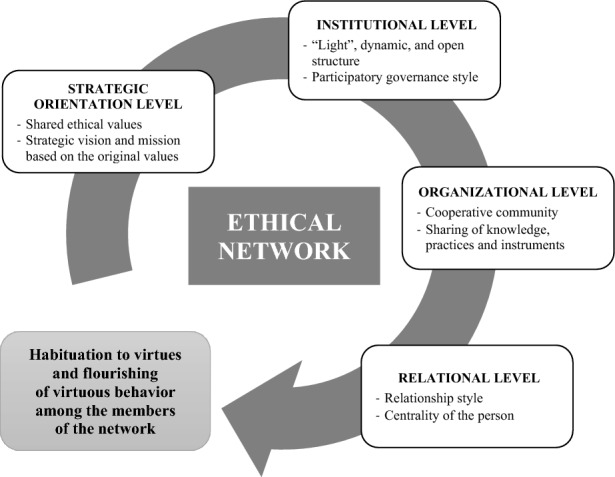
A model for development of the ethical network

The first-order terms, second-order themes, and aggregate dimensions that emerged from the study are represented in the data structure in Table 4. The data structure is a graphic representation, or ‘photograph,’ that shows how the researchers progressed from raw data terms to themes and dimensions, which helps to demonstrate rigor in qualitative research analysis (Gioia et al., 2021, p. 26). The final step in the research consisted of the grounded theory articulation. In this process, the researchers sought to identify the relationships between the emerging concepts and clarify the data-to-theory connections. Figure 1 represents the model derived from this research.

**Table 4:** Data structure

1st-Order concepts	2nd-Order themes	Aggregate dimensions
The alliance originates from common values	Shared ethical values	Strategic orientation level
The ethical substratum of ADOA was born from the strength of the charism of many founders (past and recent) who knew how to read the needs of their time and give concrete answers		
Without the commonality of values, the network could not exist as it is		
Joining the network requires an understanding of its logic and values		
Membership is not automatic, but requires that the statute is in line with the values and objectives of the network		
ADOA allows you to imagine the future rooted in the values of origin	Strategic vision and mission based on original values	
ADOA tries to understand the questions and try to share the possible answers		
The members of the network have a very specific charisma, but they need to renew it continuously in the operation		
ADOA urges us to research, evaluate and test new operational, organizational and managerial perspectives		
ADOA focuses on social needs by rooting them in the territories		
Innovating working methods frees up resources to build human relationships		
The solution to social problems (e.g., regarding young, older people or people with disabilities) comes “from the bottom” and not in an “industrial” logic		
The single organizations maintain their autonomy, even though they are part of the network	“Light,” dynamic, and open structure	Institutional level
Everyone feels free to participate within the limits of their possibilities/abilities		
The network promotes the ability of individual members to operate rather than limiting it		
The network does not have a rigid organizational structure and is based on the participation of people and organizations		
The members are held together by the commonality of values and not by strong contractual ties		
Even though there are members of very different sizes, decisions are taken in a democratic way	Participatory governance style	
The working groups (technical tables) encourage participation		
The occasions organized for discussion allow everyone to participate		
Although we share common values, we contribute as lateral thinking agents	Cooperative community	Organizational level
The heterogeneity of the organizations belonging to ADOA enriches our experiences and competences		
The comparison between different realities is cultivated		
Triggering reactions is positive because it fosters confrontation		
The diversity of activities and dimensions allows to have a critical look at the choices of the network		
A collaborative environment is built based on the idea of a shared common good	Sharing of knowledge, practices and instruments	
Feeling part of a larger community strengthens the motivation and moral behavior of workers		
Regardless of the reason for joining the network, one then learns a style of sharing and solidarity		
Participation is contagious in the style of gratuity		
Even those who are attracted by economic advantages are then contaminated by the style of sharing		
The value of gratuity is learned by practicing it in associative activities		
Enable smaller organizations to achieve sustainability		
The more structured organizations support the little ones in some phases of their development		
The less structured organizations, but more founded on volunteering, keep the original motivations alive		
The network allows access to otherwise inaccessible professionals Being part of the network helps to break the routine and rethink the way you work A managerial culture is shared		
Being supported allows us to make fewer mistakes and focus on service Establishing a community supports in difficult situations		
The network founds internal relationships on mutual trust	Relationship style	Relational level
Over time we have learned to trust each other and we also share delicate aspects of our work		
Resources, especially in terms of knowledge, are shared among the member organizations The activities are carried out with conditional reciprocity The professionals of the organizations lend their work free of charge to the network		
People are put at the center of activities Both workers and beneficiaries are considered in their dimension of persons and not in their economic role Being in the network allows us to focus our attention on fragile people, who are our reason for existing	Centrality of the person

## Findings

This section presents how ethical networks foster virtuous practices among their members. Specifically, the ethical network fosters its members’ virtuous practices on four levels, as summarized by the four aggregate dimensions that emerged from the data analysis: (1) the strategic orientation level; (2) the institutional level; (3) the organizational level, and (4) the relational level. If virtuous practices are continuously carried out within the ethical network at the four levels that emerged from this research, a habituation to virtues develops among the members of the network, as shown in Fig. 1.

### Fostering Virtuous Practices at the Strategic Orientation Level

At the strategic orientation level, two themes emerged from the data analysis: (1) shared ethical values and (2) strategic vision and mission based on the foundational values of the network. Specifically, shared ethical values represent the network’s fundamental principles and inspire its vision and mission. This occurs in a twofold way. First, Christian beliefs play an inspirational role in the ethical network regarding strategic orientation and operational activities. Second, being part of an ethical network reinforces awareness of common ethical values and the organization’s mission. For example, one interviewee stated, “For ADOA, Christian values are essential because they are the link between the members.” (I18). Another commented, “The heterogeneity of the members is valuable because it broadens the discussion, but the commonality of ethical values remains fundamental.” (I10) Yet another stated,

It would be impossible to think of ADOA without sharing the same values. We all come from the same faith and, overall, the same vision of the human person and service to frailty. Without that, it would be something else entirely, and I doubt that it could work! (I11).

Basing the organization’s strategic vision and mission on the network’s original ethical values assists sense-making because it creates a shared ethical perspective and moral vision of life and business. For example, one member declared:

Often, we are burdened by daily activities and emergencies, and we risk not having time for a fundamental aspect of our organization: our ethical roots. The ADOA helps us in the sense-making of our work... So, we can imagine and design our future without the anxiety of everyday life. (I10).

Consciousness of these fundamental values is a prerequisite to adhering to the network to ensure the possibility of building a shared ethical framework. The ADOA board analyzes all membership applications and evaluates the possibility of building positive relationships with new applicants. Entry barriers are understood as a necessary condition to preserve the network’s ethical values and primary objectives. For example, a board member affirmed:

There is no will to exclude. The ADOA remains an inclusive network. Proof of this is that we are now 38 members, while we started only with five! Nonetheless, the alliance is based on reciprocal trust and shared values. If we want to avoid any risk of bureaucratization or, worse, stiffening based on contracts and rules, we continue to encourage all candidates to engage with our logic. (I6).

With regard to the theme of strategic vision and mission based on original values, the ethical nature of the ADOA network was viewed as a guarantee of the members’ mission and an opportunity to rethink their future in light of inspirational values. Network members acknowledged that although their adhesion to the network was aimed at gaining a managerial or financial advantage, they would not have joined the network had it not been ethically oriented. In this regard, the manager of one nonprofit organization member commented, “The commonality of values is the condition *sine qua non* of our alliance.” (I9). Another explained, “Starting from common values, we can imagine the future of social services not from an industrial point of view but ‘from the bottom.’” (I16).

Interestingly, the majority of interviewees did not describe their adherence to the network in terms of economic advantage or other interests; they recognized the first contribution of the network as being the shared mission and vision in developing a reflection on Catholic social thought. The identity principle of the network is the relational bond between its members. Consequently, members desire to express and disseminate this principle to all stakeholders, particularly employees, social care service users, and local communities.

### Fostering Virtuous Practices at the Institutional Level

At the institutional level, two themes emerged from the data analysis: (1) the ‘light,’ dynamic, and open structure of the ethical network and (2) its participatory governance style.

With regard to network structure, it can be defined as ‘light’ because it encourages participation and cooperation and aims to preserve the autonomy of each member. The researchers did not detect any imposition or authoritative leadership over the member organizations by the founding institution. On the contrary, some smaller organizations found that ADOA served as an instrument to preserve their autonomy and develop their original mission. For example, a member of ADOA explained,

ADOA continuously stimulates us to improve and allows us to know other similar small organizations. In my opinion, the ADOA’s objective (and the primary motive to join the network) is not to let small organizations die and our values disperse. (I12).

With regard to the participatory governance style, ADOA does not directly deliver social care services; instead, it operates as a facilitator of relationships and synergies between its members, fostering each member’s free and spontaneous participation in common activities.

The organizational structure of ADOA is based on various discussion groups and work teams according to organizational activities, geographical area, and themes. These last, overall, were considered an important tool with which to create a cooperative mood and promote participation. As explained by one of the interviewees, the possibility to freely participate in these groups is one of the core characteristics of ADOA: “The technical round tables make ADOA’s operations immediate and involve as many representatives as possible from each new organization that adheres to the alliance.” (I18).

### Fostering Virtuous Practices at the Organizational Level

At the organizational level, two themes emerged from the data analysis: (1) cooperative community of the network and (2) sharing of knowledge, practices, and instruments.

With regard to the first theme, the explicit ethics-driven purpose of the network plays a crucial role in fostering consistent behaviors from its members, whose primary mission is mutual service. For example, one interviewee explained how the goal of the network is fundamental in concretizing the common good into practical action:

The concept of solidarity, dear to Catholic social thought, goes beyond some sporadic initiative. It implies a firm and persevering commitment to the common good and the acknowledgment of mutual responsibility... By pursuing the good of its community, the ADOA is trying to create a collaborative environment where each participant contributes their best. (I8).

The importance of the cooperative community of the network emerged in particular in the context of the COVID-19 pandemic:

I experienced this first-hand during the COVID-19 pandemic: being in a group with other organizations made it possible to really give each other great help, in a very dangerous period, with risks of all kinds, even from a legal point of view. (I22).

With regard to the sharing of knowledge, practices, and instruments, an important aspect is the gratuitousness of individual contributions based on relational reciprocity. People are encouraged to contribute by experimenting with the nature of relations among network members:

Some of our members were attracted by the possibility of getting advantages from their adhesion to the ADOA. Once inside the community, they understood the value of gratuitousness, carried out by example, and paradoxically they got more benefits than if they had to pay for them! (I1).

Mutual aid can be realized by the free, voluntary, and gratuitous contributions of members, such as by offering professional help to specific individuals in particular circumstances (e.g., during the COVID-19 pandemic crisis) or by participating in work teams and sharing their experiences. One interviewed explained, “Belonging to the network has stimulated us to seek comparison with other experiences and relating to each other, making the most of the resources we have.” (I17).

This model of participation makes possible a continuous exchange of experiences, which incidentally facilitates a collaborative work style. The data analysis made clear that by participating in ADOA’s activities, members formed a habit of sharing their competencies and capacities in the name of solidarity and gratuitousness rather than conditional reciprocity. Several interviewees underlined that ADOA created a relational environment where they felt free to contribute without competitive pressures or performance anxiety. One interviewee affirmed, “The fact that there are scheduled appointments allows you to really collaborate with others, and change is also only by ‘osmosis’... and even if there is no change, divergent thinking emerges.” (I18).

The network prioritizes values such as gratuitousness, solidarity, honesty, and trust over individual merit. While individual competencies are recognized, they are considered common resources that can be shared rather than a measure of a competitor’s strength. This relational style affects behaviors during network meetings and daily working life. Two different perspectives on the empowerment of managers and workers emerged from the interviews. One interviewee declared, “We need to continuously remind our employees and volunteers about our original purpose of social helping. ADOA contributes to our efforts by reflecting on the updating of values such as charity or solidarity in the contemporary scenario.” (I11). Another stated, “The climate that reigns in ADOA affects our workers, who are more inclined to collaborate with each other.” (I9).

### Fostering Virtuous Practices at the Relational Level

At the relational level, two themes emerged from the data analysis: (1) relational style of the network and (2) centrality of the person. Specifically, the relational style of the network has led members to develop a nonutilitarian relationship based on relational reciprocity. Trust is key in the relational style between managers and workers of ADOA’s member organizations. Trust makes it possible to overcome competition and is grounded in common values and the habit of sharing knowledge, competencies, and experiences. One interviewee stated, “During the years, we had the opportunity to deepen our reciprocal trust, and now we discuss several managerial tasks with other ADOA members, such as normative updating or the adoption of new working/technological methods.” (I2).

Although ADOA’s members provide social and health services, they experiment with competition, leading to an increased risk of building a competitive work environment. The network serves as a sort of gymnasium, where managers and workers are encouraged to abandon their daily concerns and build trustful and cooperative relationships. This type of relationship is also mirrored in individual organizations. As one interviewee noted, many workers participate in the network’s activities and are more collaborative when they come back to their organization. Another stressed the effect on individual behavior and motivations: “ADOA helps build an environment, a style... so people become better too. If the environment does not communicate and encourage these values, one tends to cater to the lowest common denominator.” (I22).

Further, by experimenting with the ethical network, people are also encouraged to reflect on their work’s moral dimension:

The relationship is not only between entities but also between people: it helps entities that do not have the opportunity to see/think about new things... but also causes doubts in those who always go their own way, maybe making them also reflect on the ethical dimension of what they do. (I17).

Finally, ADOA promotes a caring culture and fosters positive relationships between employees and service beneficiaries (e.g., older people or people with disabilities or other needs), who are seen for their potentialities rather than their limitations. The centrality of the person was highlighted by one interviewee, who stated: “ADOA’s goal is to promote a new style of caring: a culture of meeting and engagement, and an attempt to build relationships that are good and constructive.” (I21).

### Toward a Circular Model of Virtuousness of Ethical Networks

The findings show that ADOA is capable of fostering virtuous practices among its members and is, therefore, a virtuous ethical network. By adhering to and practicing in the network, members develop a habit ‘for the good’; that is, a habituation to act virtuously. They develop character virtues such as gratuitousness, solidarity, and honesty because they practice ‘good’ relationships and are also encouraged to practice virtues in their individual organizations.

Further, by sharing their experiences and competencies and aspiring to the same ethical values, network members develop the attitude of deciding wisely; that is, making decisions for the common good of people, organizations, and society. Members cultivate a common managerial style inspired by cooperation and trust, thus experience virtuous practice, accumulate social capital, and flourish in virtues. This was highlighted by some interviewees: “Mirroring other experiences and observing good practices make us capable of improving our practices and of doing better what we daily do.” (I15).

In the various difficulties we face daily, we often lack the time and serenity to discern decisions. In ADOA, we find a place where we can grow our ability to do the right thing and preserve the ‘legacy of good’ that has been entrusted to us. (I19).

The network encourages divergent thinking and allows members to interpret their reality, not for reasons of organizational efficiency but to promote ethical values and common good goals. In other words, ADOA fosters a critical view of organizational and managerial problems in which the moral dimension of choices is prominent.

As mentioned above, good practices are experienced in the network but are also exported to individual organizations through osmosis. People who practice ‘good’ are also capable of practicing it in their organizations, as noted by a member of the network: “The number of workers from my organization participating in ADOA’s meetings has increased over the years. Gradually they learn a style of working, sharing, and reflecting on problems... and all that silently improves our daily activities.” (I9).

Thus, the ethical network fosters a habituation to and the propagation of virtuous behaviors among its members at the four levels, as represented in the circular model illustrated in Fig. 1. More precisely, establishing an ethical network based on the abovementioned four levels of virtuous behaviors helps organizations and individuals flourish in virtues and habituate toward virtuous practices, compelling them to pursue their mission, preserve their values, and strive for moral and business excellence.

## Discussion

This study investigated how ethical networks promote virtuous practices among their associated nonprofit organizations. Based on a grounded theory approach, the analysis revealed that networks can not only be virtuous themselves, as previously emphasized by Vriens et al. (2018), but also foster virtuous practices among their members at four different (but complementary) levels. Specifically, this study highlights that ethical networks foster virtuous practices on multidimensional levels, including strategic orientation, institutional, organizational, and relational levels.

With regard to fostering virtuous practices at the strategic orientation level, the results of this study support Silvestri and Veltri’s (2017) assertion on the importance of ethical values, namely that they are at the origin of an ethical network. This research highlights the importance of aligning ethical values between the network and its members in such a way that all actual and potential members of an ethical network share ethical values to foster virtuous practices within the network itself. More precisely, this study suggests that shared ethical values such as a common religious belief serve as the network’s ‘glue.’ Further, members of the ethical network understand their participation as an encouragement to safeguard, cultivate, and reinforce their ethical mission. Indeed, in facing their daily challenges, nonprofit organizations understand that staying grounded in their values is an unavoidable condition for their survival and development. They stay in the alliance because of an ethical coherence. While previous studies (e.g., Burchielli et al., 2009) have focused on the normative role of ethical networks by showing how they provide common ethical rules to their participants, this study emphasizes the sharing of values and coherent virtuous behaviors as the core point of the ethical network.

At the institutional level, ethical networks foster virtuous practices among their members by focusing on democratic participation rather than on a hierarchy, enabling all members to take part in the network’s activities and ignoring standard market elements such as relative size or negotiating power. This aspect of network governance emerges as essential in case of an ethical network promoted by a powerful entity such as the Catholic Church. Further, the choice of a private (not canonic) juridical form and a governance model that preserves the decisional autonomy of the members is fundamental to protecting the network from risk of external control or, worse, abuse. Although substantial functionality is essential to defining the ethical nature of the network, the formal/juridical structure also appears important to creating an institution in which good practices can be experienced. Moreover, this study outlines the importance of having an inclusive organizational model by providing formal and informal places of engagement for the largest possible number of managers and workers according to personal competencies and interests. Finally, another essential institutional element is the guarantee of individual autonomy of organizational members of the network. Both large and small organizations fully preserve their decisional and patrimonial autonomy. The network is where they can improve their decisional capacity, enforce their ethical missions, and test a way to work; however, they are called to make good decisions themselves.

At the organizational level, even if the formal structure of the network is essential to creating the conditions for an effective operation, members experience good practices only if such relationships are put in place, as Cameron et al. (2011) argued. In accordance with this previous research, this study highlights that the network fosters the gratuitous exchange of competencies, experiences, and capabilities so that members cultivate social capital and experiment with a virtuous way to collaborate. Cooperation, sharing, trustworthiness, and gratuitousness become the pillars of the ethical network if members are encouraged to participate and engage in mutual aid.

With respect to fostering virtuous practices at the relational level, this research shows that an ethical network develops through a relational environment in which people build relationships as people and as professionals. In this sense, the ethical network becomes a ‘community of practice,’ where people experience a way of working based on values such as gratuitousness, trust, loyalty, and solidarity. Overcoming the organizational level of the ethical network allows for individual virtues and a relational style between individual member organizations to flourish. Virtuous interpersonal relationships are mirrored in a reinforced culture of care, where disadvantaged people (e.g., older people or those with disabilities or experiencing financial hardship) are at the very center of activities as people with needs, emotions, and feelings. At the same time, the promotion of a nonutilitarian way of building relationships facilitates the creation of ethical working environments, preventing and limiting potential tensions or conflicts of interests, as previously shown by Santana et al. (2009).

The four aggregate dimensions that emerged from the analysis are all essential to develop the ethical network and are encapsulated in the circular model shown in Fig. 1. The analysis shows that no dimension prevails over or is more important than the others. Each contributes equally to fostering virtuous practices for the development of the ethical network. The habituation to virtues and the flourishing of virtuous behaviors among network members are not simply added bonuses; rather, they are crucial elements that serve to continuously promote the virtuous practices at each of the four levels to develop the network itself. Although these findings have emerged from a specific context, they represent “portable principles” (Gioia et al., 2013) that are transferable to other ethical networks.

## Theoretical Implications

The findings of this research contribute in multiple ways to advancing the knowledge on ethical networks by showing that virtuous ethical networks can foster the flourishing of virtues by promoting ethical practices among their members at different levels. First, while previous studies have focused on the characteristics of ethical networks (Burchielli et al., 2009) and the union of value-driven organizations with a shared ethical commitment (Silvestri & Veltri, 2017; Vaccaro, 2012), this research provides an original contribution to the literature about the virtuousness of ethical networks by showing that being part of a network encourages ethical behavior and enforces a commitment to common objectives, as affirmed by Haney (2017). Further, even though this study focused on an ethical network as an alliance rooted in a common ethical framework, it shows that a network can be a virtuous structure in the sense proposed by Vriens et al. (2018).

Second, previous studies (e.g., Sison & Fontrodona, 2013) have affirmed that an organization (in this case, an ethical network) based on shared values fosters individual participation without limiting contributions to the terms of a contract. This study supports this idea by highlighting that common ethical values, if clearly defined when an organization joins the network as well as during its daily activities, allows the development of a robust ethical network and the flourishing of virtuous practices. Consequently, members of the network are aware of the common ethical framework and act consistently with it. In this sense, this study empirically validates the first condition for realizing a virtuous structure, the so-called teleological context (Vriens et al., 2018), in line with authors that connect organizational virtuousness with the main goal of the organization (Cameron et al., 2004). Overall, the study confirms that a common ethical framework and a shared purpose are at the heart of the ethical network and overcome individual interests, as previous studies have highlighted regarding the network approach to business ethics (e.g., Floridi, 2009).

Third, this study not only indirectly confirms the three contexts that virtuous networks provide to their members (as in Vriens et al., 2018) but also makes an original contribution by introducing the institutional level of the network. The results highlight that the institutional design of the network is essential to define it as a virtuous structure. Further, the characteristics theoretically proposed by Vriens et al. (2018) are confirmed by adding new empirical knowledge about how networks foster virtuous practices among their members. Indeed, the data analysis shows that to become an institution that fosters good practices—in line with the definition by MacIntyre (1985)—there is a need for structural elements such as democratic governance, places of engagement for workers, and individual autonomy. These elements may be considered innovative and positively contribute to the scientific debate on ethical networks. This study underlines that the virtuousness of an ethical network is linked to its institutional structure, even though—as affirmed by previous authors (Baucus & Beck-Dudley, 2005; Cameron et al., 2004)—an organization can be considered virtuous based on its concrete impact on individual and community practices. In contrast, the study suggests that an ethical network cannot simply be considered virtuous based on the virtuousness of its members but rather if it cultivates and exports virtues to each organizational member. In other words, an ethical network is only such if it demonstrates the capacity to foster individual virtues and, further, to make possible the enforcement of organizational virtues of its members. This finding seems to partially support the intrinsic hypothesis by Bright et al. (2014) on organizational virtuousness, although synergistic actions were also demonstrated.

Moreover, this study was conducted following the outbreak of COVID-19, which created several tensions in healthcare organizations, confirming the buffering effect of virtuousness for ethical networks, as suggested by previous studies (Bright et al., 2006; Caza et al., 2004). This buffering effect was empirically validated given that a common effort based on solidarity and gratuitousness helped to overcome the potential adverse effects of the pandemic, which other organizations based on procedures, rules, or economic incentives were unable to replicate.

Finally, this study contributes to the development of a descriptive/empirical approach to the study of organizational virtuousness. Indeed, past studies have addressed this topic mainly in normative terms, which is common in virtue-based business ethics. In contrast, this study adopted the case study method and a grounded approach to understand the effects of membership to an ethical network in terms of the flourishing of virtuous practices, thus providing a new perspective on the connection between normative and descriptive research in business ethics. This contribution should be explored in future research.

## Practical and Social Implications

This study proposes a model by which ethical networks foster virtuous practices among their members. These findings may be beneficial to both ethical network governance and management as well as individual organizations willing to reinforce their values in the pursuit of their mission. By fostering reciprocity and relationships based on mutual assistance, the ethical network positively influences its members and enables them to enact good practices. An ethical network contributes to creating social/relational capital and facilitates its members to cultivate their own social/relational capital. An ethical network creates a virtuous environment by focusing on values and trust rather than on contracts and interests, thereby creating a context within which members can continuously focus on their purpose and maintain their core values. Although the ethical network does not directly act as a service provider, it promotes reciprocity, gratuitousness, and trust among its members. Therefore, the ethical network serves as an educational environment in which individuals and organizations can cultivate themselves without pursuing their own immediate interests. The match between the ethical roots and practice of virtues enables organizations and individuals to create experiences from virtue-based behaviors and develop practical wisdom. Participating in an ethical network helps individual organizations face the complexity of the relational and competitive environment of the social care sector by strengthening their awareness of their social role, ethical values, and purpose. The competitive environment involves dealing with new for-profit competitors and building new relationships with public administrators in an uncertain regulatory and financial context. Consistently, an ethical network can foster virtues if it becomes a community of practice in the MacIntyrian sense (Moore, 2012b), where the relational style is based on ethical values. The network becomes the perfect place for good practices to be developed—members are encouraged to participate by doing their best in terms of gratuitousness and solidarity but also professionalism, such as by building thematic worktables and sectorial areas to allow individual contributions in line with the attitudes, preferences, and abilities of each member. To realize this goal, the network incentivizes broad participation that is not limited to top managers or board members but extended to middle managers and other workers. Finally, the study underlines how an ethical network does not stop at the institutional level but facilitates interpersonal relationships. Institutions are important as environments for practicing good, but the individual level of virtuousness requires that each person involved in the network (and the largest number of people) can experiment with virtues in action.

The findings of this research also have social implications. First, virtuous networks, organizations, and individuals can compensate for the lack of or inefficiency of public services, especially in times of crisis, thus benefit society by taking care of the community’s well-being. For example, the virtuous behavior of organizations and individuals was shown to be crucial during the COVID-19 pandemic, in which extraordinary efforts were required to overcome the health, social, and economic crisis. Second, through their practices, virtuous networks can also inspire other nonprofit and for-profit organizations and networks to pursue their mission in a virtuous way, with positive repercussions for individuals working in these organizations and, more broadly, for society.

## Conclusion

The contemporary social and economic environment calls for a broader reflection on organizational models that can foster the development of virtues. This paper focused on an ethical network in the social care sector as a case study, providing a possible model for an effective virtuous structure based on four dimensions. The research has demonstrated how shared ethical values and religious beliefs affect network building and effectiveness, together with consistent organizational and institutional choices. The consciousness of the ethical mission is at the heart of network management, even if there is also a need to continuously nurture the relationship between members to strengthen the network and avoid conflicts of interest. Further, the evidence provided by the case study supports the idea that virtuous structures enable members to develop virtues—above all, practical wisdom—and improve moral behaviors in workplaces.

The present research has some limitations that could provide an opportunity for further research. First, although the evidence provided is solid, it is limited to a specific case study with characteristics such as homogeneity of members operating in the same economic sector (social care services), a circumscribed area, and the religious nature of shared values. Further research could be conducted on other ethical networks that do not have an ethical framework connected by religious beliefs and principles or that operate across different economic fields. For example, the ethical network framework could be applied to other national or international organizations such as the Economy of Communion. Further, future research could focus on ethical networks in the business field. Second, this research focused on the network itself and perceptions of its effectiveness by managers, board members, and other people in positions of responsibility of member organizations. Future research could include the perceptions of other workers involved in service provision as well as external stakeholders to evaluate the pervasiveness of the effects of the network on practicing the virtues. Finally, it would be interesting to conduct a longitudinal study to understand how the network and the internal relationships among the members evolve.
